# Genomic Regions Associated With Skeletal Type Traits in Beef and Dairy Cattle Are Common to Regions Associated With Carcass Traits, Feed Intake and Calving Difficulty

**DOI:** 10.3389/fgene.2020.00020

**Published:** 2020-02-04

**Authors:** Jennifer L. Doyle, Donagh P. Berry, Roel F. Veerkamp, Tara R. Carthy, Siobhan W. Walsh, Ross D. Evans, Deirdre C. Purfield

**Affiliations:** ^1^Animal and Grassland Research and Innovation Centre, Moorepark, Teagasc, Fermoy, Ireland; ^2^Department of Science, Waterford Institute of Technology, Waterford, Ireland; ^3^Animal Breeding and Genomics Centre, Wageningen Livestock Research, Wageningen University & Research, Wageningen, Netherlands; ^4^Irish Cattle Breeding Federation, Bandon, Ireland

**Keywords:** cattle, genome-wide association study, linear type traits, single nucleotide polymorphism, skeletal, sequence

## Abstract

Linear type traits describing the skeletal characteristics of an animal are moderately to strongly genetically correlated with a range of other performance traits in cattle including feed intake, reproduction traits and carcass merit; thus, type traits could also provide useful insights into the morphological differences among animals underpinning phenotypic differences in these complex traits. The objective of the present study was to identify genomic regions associated with five subjectively scored skeletal linear traits, to determine if these associated regions are common in multiple beef and dairy breeds, and also to determine if these regions overlap with those proposed elsewhere to be associated with correlated performance traits. Analyses were carried out using linear mixed models on imputed whole genome sequence data separately in 1,444 Angus, 1,129 Hereford, 6,433 Charolais, 8,745 Limousin, 1,698 Simmental, and 4,494 Holstein-Friesian cattle, all scored for the linear type traits. There was, on average, 18 months difference in age at assessment of the beef versus the dairy animals. While the majority of the identified quantitative trait loci (QTL), and thus genes, were both trait-specific and breed-specific, a large-effect pleiotropic QTL on BTA6 containing the *NCAPG* and *LCORL* genes was associated with all skeletal traits in the Limousin population and with wither height in the Angus. Other than that, little overlap existed in detected QTLs for the skeletal type traits in the other breeds. Only two QTLs overlapped the beef and dairy breeds; both QTLs were located on BTA5 and were associated with height in both the Angus and the Holstein-Friesian, despite the difference in age at assessment. Several detected QTLs in the present study overlapped with QTLs documented elsewhere that are associated with carcass traits, feed intake, and calving difficulty. While most breeding programs select for the macro-traits like carcass weight, carcass conformation, and feed intake, the higher degree of granularity with selection on the individual linear type traits in a multi-trait index underpinning the macro-level goal traits, presents an opportunity to help resolve genetic antagonisms among morphological traits in the pursuit of the animal with optimum performance metrics.

## Introduction

Linear type traits have been used in both beef and dairy cattle since the early 20th century to characterize the skeletal characteristics of an animal (Berry et al., 2019). These type traits have previously been identified as being moderately to strongly genetically correlated with a range of performance traits in cattle including feed intake (Veerkamp and Brotherstone, 1997; Crowley et al., 2011), reproductive traits (Berry et al., 2004; Wall et al., 2005; Carthy et al., 2016), carcass merit (Mukai et al., 1995; Berry et al., 2019), animal value (Mc Hugh et al., 2010), and health (Ring et al., 2018). As type trait measurements are typically taken when an animal is young (Doyle et al., 2018), they may be useful as early predictors of the correlated traits which are often measured later in life or after the animal is slaughtered. While type traits are also moderately to strongly correlated with live-weight (Mc Hugh et al., 2010; Berry et al., 2019) and carcass weight (Conroy et al., 2010), none of these correlations are unity, implying that two animals with the same weight may be morphologically very different; for example, a tall animal with a short back may have the same (carcass) weight as a short animal with a long back. Therefore, including linear type traits in future genetic and genomic evaluations as part of a multi-trait evaluation including also the goal trait of interest may provide additional information over and above what could be gleaned from the goal trait alone.

While many genomic studies have been carried out on stature in both beef and dairy cattle (Pryce et al., 2011; Bolormaa et al., 2014), few studies have been published on the underlying genomic features contributing to differences in other skeletal linear type traits in either beef (Vallée et al., 2016) or dairy (Cole et al., 2011; Wu et al., 2013; Sahana et al., 2015) cattle. No previous study has attempted to identify quantitative trait loci (QTL) associated with the skeletal traits in multiple breeds or to compare and contrast detected QTLs to previously identified QTLs associated with correlated complex phenotypes such as carcass merit, feed intake and efficiency, and calving performance. Therefore, the objective of the present study was to identify genomic regions associated with five subjectively scored skeletal linear traits to determine if these associated regions are common in multiple beef and dairy breeds and also to determine if these regions overlapped with previously identified QTLs associated with other correlated performance traits.

## Materials and Methods

Animal Care and Use Committee approval was not obtained for the present study as the data were obtained from the existing Irish Cattle Breeding Federation (ICBF) national database^1^.

### Beef Phenotypes

Routine scoring of linear type traits is carried out on both registered and commercial beef herds by trained classifiers from the Irish Cattle Breeding Federation as part of the Irish national beef breeding program (Mc Hugh et al., 2010; Berry and Evans, 2014). Five skeletal type traits scored on a scale of 1 to 10 on beef cattle describing the wither height (WH), back length (BL), chest depth (CD), chest width (CW), and hip width (HW) were included for analysis in the present study (Supplementary Table S1). Data on these linear type traits were available on 147,704 purebred Angus (AA), Charolais (CH), Hereford (HE), Limousin (LM), or Simmental (SI) beef cattle, all scored between the ages of 6 and 16 months between the years 2000 and 2016, with only one (i.e., the first) record per animal retained.

Animals were discarded from the dataset if the sire, dam, herd, or classifier was unknown. Only data from classifiers that scored ≥ 100 animals since the year 2000 were kept. Animals were also discarded from the dataset if the parity of the dam was unknown; parity of the dam was subsequently recoded into 1, 2, 3, 4, and ≥ 5. Contemporary group was defined as herd-by-scoring date generated separately per breed. Each contemporary group had to have at least five records. Following edits, data were available on 81,200 animals, aged between 6 and 16 months, consisting of 3,356 AA, 31,049 CH, 3,004 HE, 35,159 LM, and 8,632 SI.

### Dairy Phenotypes

Scoring of linear type traits in the Irish dairy herd is undertaken by trained classifiers from the Irish Holstein-Friesian Association (Berry et al., 2004). For the purpose of the present study, three skeletal linear type traits that closely align to one of the five beef skeletal traits were selected for analysis. These traits were stature (STA which is comparable to WH in beef), rump width (RW which is comparable to HW in beef), and chest width (CWD which is comparable to CW in beef). In dairy cattle, these traits were scored on a scale of 1 to 9 (Supplementary Table S2) with the direction of scale the same as the comparable traits in the beef herd. Linear type trait information on 239,776 first parity cows was available between the years 2000 and 2016; only the first record per cow was retained.

Animals were discarded from the dataset if the sire, dam, herd, or classifier was unknown. Records were also discarded from the data set if scored after 10 months of lactation. Only data from classifiers that scored > 100 animals since the year 2000 were retained. Contemporary group was defined as herd-by-scoring date and each contemporary group had to have at least five records. Following edits, data were available on 117,151 primiparous Holstein-Friesian cows (HF) aged between 23 and 42 months at scoring.

### Generation of Adjusted Phenotypes

Prior to inclusion in the analysis, all beef cattle phenotypes were adjusted, within breed, in ASREML (Gilmour et al., 2009) using the model:

*y*_ijklm_ = μ + HSD*_*m*_* + Sex*_*j*_* + AM*_*k*_* + DP_l_ + animal*_*i*_* + *e*_ijklm_

where *y*_ijklm_ is the linear type trait, μ represents the population mean, HSD*_*m*_* is the fixed effect of herd-by-scoring date (*m* = 11,130 levels), Sex*_*j*_* is jth sex of the animal (male or female), AM*_*k*_* is the fixed effect of the age in months of the animal (*k* = 11 classes from 6 to 16 months), DP_l_ is the fixed effect of the parity of the dam (*l* = 1, 2, 3, 4, and ≥5), animal*_*i*_* is the random additive effect of animal *i*, and *e*_ijklm_ is the random residual effect. The adjusted phenotype was the raw phenotype less the fixed effect solutions of HSD, Sex, AM, and DP.

The dairy phenotypes were also adjusted in ASREML (Gilmour et al., 2009) using the model:

*y*_ijklm_ = μ + HSD*_*m*_* + AM*_*j*_* + CM*_*k*_* + LS_*l*_ + animal*_*i*_* + *e*_ijklm_

where *y*_ijklm_ is the linear type trait, μ represents the population mean, HSD*_*m*_* is the fixed effect of herd-by-scoring date (*m* = 9,591 levels), AM*_*j*_* is the fixed effect of the age in months of the animal at scoring (*j* = 20 levels from 23 to 42 months), CM*_*k*_* is the fixed effect of the month of calving (*k* = 12 levels from 1 to 12), LS*_*l*_* if the fixed effect of the stage of lactation of the animals (*l* = 10 levels from 1 to 10 reflecting number of months of lactation), animal*_*i*_* is the random additive effect of animal *i*, and *e*_ijklm_ is the random residual effect. The adjusted phenotype was the raw phenotype less the fixed effect solutions of HSD, AM, CM, and LS.

### Genotype Data

Of the edited dataset of 81,200 beef animals and 117,151 dairy animals with linear type trait information, 23,943 animals from six breeds (1,444 AA, 6,433 CH, 1,129 HE, 8,745 LM, 1,698 SI, and 4,494 HF) also had genotype information available. These genotypes were imputed to whole genome sequence (WGS) as part of a larger dataset of 638,662 genotyped animals from multiple breeds as detailed by Purfield et al. (2019). All 638,662 genotyped animals were genotyped using either the Bovine Illumina SNP50 (*n* = 5,808; 54,001 SNPs), the Illumina High Density (HD; *n* = 5,504; 777,972 SNPs), the Illumina 3k panel (*n* = 2,256, 2,900 SNPs), the Illumina LD genotyping panel (*n* = 15,107, 6,909 SNPs) or a bespoke genotype panel (IDB) developed in Ireland (Mullen et al., 2013) which was either on version 1 (*n* = 28,288; 17,137 SNPs), version 2 (*n* = 147,235; 18,004 SNPs) or version 3 (*n* = 434,464; 53,450 SNPs). Each animal had a call rate ≥ 90%. Only autosomal SNPs, SNPs with a call rate ≥ 90% and those with a known chromosome and position on UMD 3.1 were retained for imputation.

Imputation to HD was carried out on all genotyped animals using a two-step approach in FImpute2 with pedigree information (Sargolzaei et al., 2014); this involved imputing animals genotyped on the 3k, LD, or IDB panels to the Bovine SNP50 density and subsequently imputing all resulting genotypes (including the Bovine SNP50 genotypes) to HD using a multi-breed reference population of 5,504 influential sires genotyped on the HD panel. Imputation to WGS was then undertaken using a reference population of 2,333 *Bos Taurus* animals of multiple breeds from Run6.0 of the 1000 Bulls Genomes Project by first phasing all 638,662 imputed HD genotypes using Eagle (version 2.3.2; Loh et al., 2016) and subsequently imputing to WGS using minimac3 (Das et al., 2016).

Quality control edits were imposed on the imputed sequence genotypes within each of the six breeds separately; all SNPs with a minor allele frequency (MAF) ≤ 0.002 were removed and regions of poor WGS imputation accuracy, identified using 147,309 verified parent-progeny relationships as previously described by Purfield et al. (2019), were then removed. Following all SNP edits, 16,342,970, 17,733,147, 16,638,022, 17,803,135, 17,762,681, and 15,542,919 autosomal SNPs remained for analysis in the AA, CH, HE, LM, SI and HF populations, respectively.

### Association Analyses

The association analyses were performed, within each breed separately, using a mixed linear model in Genome-wide Complex Trait Analysis (GCTA) (Yang et al., 2011). Autosomal SNPs from the original HD density panel (i.e., 734,159 SNPs) were used to construct the genomic relationship matrix (Yang et al., 2010). The model used for the within-breed analysis was:

y=μ+xb+u+e

where **y** is a vector of preadjusted phenotypes, μ is the overall mean, **x** is the vector of imputed genotypes, **b** is the additive fixed effect of the candidate SNP to be tested for association, **u** ∼ N(0,**G$\sigma_{\text{u}}^{2}$**) is the vector of additive genetic effects, where **G** is the genomic relationship matrix calculated from the imputed HD SNP genotypes, and $\sigma_{u}^{2}$ is the additive genetic variance, and **e** ∼ N(0,**I$\sigma_{\text{e}}^{2}$**) is the vector of random residual effects, with **I** representing the identity matrix and $\sigma_{e}^{2}$ the residual variance.

### QTL Detection, Gene Annotation, and Variance Explained

A significance threshold of *p* ≤ 1 × 10^–8^ and a suggestive threshold of *p* ≤ 1 × 10^–5^ was applied genome-wide for each SNP in each trait as per Wang et al. (2016). Significant and/or suggestive SNPs that were within 500 kb of each other were classed as being within the same QTL. Genes within these QTLs were then identified using Ensembl 94 (Zerbino et al., 2017) on the UMD 3.1 bovine genome assembly. Cattle QTLdb^2^ was used to identify if any of the QTLs identified within the present study had previously been associated with any other traits in beef or dairy cattle. To identify QTL regions that were suggestive in more than 1 breed, each chromosome was split into 1 kb genomic windows and windows containing suggestive SNPs (*p* ≤ 1 × 10^–5^) were compared across the breeds.

The proportion of genetic variance of a trait explained by a SNP was calculated as:

$$\frac{2\,p\,\left(1-p\right)\,a^{2}}{\sigma_{g}^{2}}$$

where *p* is the frequency of the minor allele,*a* is the allele substitution effect and $\sigma_{g}^{2}$ is the genetic variance of the trait in question as calculated from the association analyses.

### Meta-Analyses

Following the within breed analyses, meta-analyses were conducted for CD and BL across the five beef breeds and for WH, CW, and HW across all six breeds using the weighted *Z*-score method in METAL (Willer et al., 2010). METAL uses the *p*-values and the direction of SNP effects from the individual analysis and weights the individual studies based on the sample size to calculate an overall *Z*-score:

$$Z=\frac{{\text{Σ}}_{i}\,z_{i}\,w_{i}}{\sqrt{{\text{Σ}}_{i}\,w_{i}^{2}}}$$

where *w* is the square root of the sample size of the *i*th breed, and *z* is the *z*-score for the *i*th breed calculated as *z*_*i*_ = ϕ^(−1)^ (1−*P*_*i*_/2)Δ_*i*_, where Φ is the cumulative distribution function, and P_*i*_ and Δ_*i*_ are the *P*-value and direction of effect for breed *i*, respectively.

### Enrichment Analyses

Enrichment analysis was carried out among all suggestive and significant SNPs within each trait and each breed separately to estimate if the number of SNPs in each annotation class was greater than what would be expected by chance (Bouwman et al., 2018):

$$\text{enrichment}\,\text{=}\,\frac{a}{b}\,{\left[\frac{c}{d}\right]}^{-1}$$

where *a* is the number of suggestive and/or significant SNPs in the annotation class of interest, *b* is the total number of suggestive and/or significant SNPs that were associated, *c* is the total number of SNPs in the annotation class in the association analysis, and *d* is the overall number of SNPs included in the association analysis.

## Results

The scale of measurement, number of records, mean, and standard deviation of the linear type traits in each breed is in the Supplementary Tables S1, S2. The average age of the beef cattle at measurement was 10 months, while the average age of the dairy cows was 28 months; hence there was, on average, an 18-month difference in age at classification between the dairy and beef populations. Significant (*p* ≤ 1 × 10^–8^) and/or suggestive (*p* ≤ 1 × 10^–5^) SNPs were detected for all of the traits in all six breeds; however, the exact locations of these SNPS, and the direction of the effects of these SNPs, differed by breed.

### Wither Height/Stature

No 1 kb genomic window associated with height was common to all six breeds. There was, however, some overlap in suggestive 1 kb windows between the AA and LM where 79 suggestive windows located on BTA6 were common to both breeds (Supplementary Figure S1). Six genes were identified within these windows on BTA6 including *NCAPG* and *LCORL*. There were also two suggestive 1 kb windows located at approximately 94.9 Mb on BTA5 common to both the AA and HF.

The strongest associations in both the AA and LM were intergenic variants located in QTLs surrounding the *NCAPG* and *LCORL* genes on BTA6 (Table 1 and Supplementary Figure S2) and accounted for 0.6 and 0.04% of the genetic variation in WH in the AA and LM, respectively. Five intronic variants and three downstream gene variants located within the *LCORL* gene, and 12 intronic variants located within the *NCAPG* gene, were suggestively associated in the AA (*p* < 9.18 × 10^–6^) and significantly associated in the LM (*p* < 1.29 × 10^–12^). Interestingly, the positive (i.e., taller) allele of these SNPs occurred at similar frequencies (0.08–0.09) in both the AA and LM and had a similar effect size in both breeds. In comparison, while these SNPs were segregating in both the HE and HF, and had similar allele frequencies in the HE as in the AA and LM, none of these SNPs were near significance in either the HE (*p* > 0.11) or HF (*p* > 0.88). However, a suggestive association was detected 21 Mb further upstream of *LCORL* on BTA6 in the HF where the strongest association within this QTL, rs209851496 (*p* = 1.94 × 10^–6^), was located 1kb upstream of the *CHRNA9* gene.

**TABLE 1:** The location of the most significant QTLs, limited to the top 5, which were associated with wither height or stature, and the genes located within these QTLs within each breed.

				**No of suggestive and significant SNPs**	**Most significant SNP**		**Allele frequency of positive allele**						
								
**Breed**	**Chr**	**Start**	**End**			***P*-Value**	**AA**	**CH**	**HE**	**LM**	**SI**	**HF**	**Candidate genes within this QTL**
Angus	6	37859028	40529961	96	39955422^a^	7.31 × 10^–9^	0.114	0.000	0.000	0.064	0.000	0.042	ABCG2, PKD2, SPP1, MEPE, LAP3, NCAPG*, LCORL*
	6	40760106	41784760	14	41276346^b^	2.74 × 10^–7^	0.445	0.000	0.372	0.522	0.000	0.784	SLIT2*, PACRGL, KCNIP4
	16	72342264	73978632	25	72877647^a^	1.46 × 10^–7^	0.995	0.002	0.003	0.000	0.996	0.996	RPS6KC1, BATF3, PPP2R5A*
	20	46866355	47884741	51	47372538^a^	7.48 × 10^–7^	0.161	0.310	0.523	0.768	0.822	0.834	ENSBTAG00000048105
	26	40278450	41826296	23	41323903^c^	2.21 × 10^–7^	0.993	0.980	0.982	0.017	0.983	0.000	WDR11*, PTPRG, FHIT
Charolais	2	5346602	6349651	2	5846602^a^	6.02 × 10^–8^	0.690	0.585	0.703	0.000	0.586	0.390	NAB1, MSTN, MFSD6
	5	40455760	41765149	12	40955760^a^	5.68 × 10^–8^	0.000	0.038	0.987	0.000	0.016	0.010	SLC2A13*, ABCD2
	6	33942529	35471763	9	34442529^a^	7.78 × 10^–6^	0.998	0.011	0.000	0.003	0.000	0.000	CCSER1
	27	11896148	12929004	15	12428578^a^	7.97 × 10^–7^	0.295	0.464	0.000	0.513	0.501	0.344	TENM3, DCTD
	28	11615130	12630615	8	12127037^a^	6.97 × 10^–7^	0.758	0.975	0.273	0.138	0.153	0.146	
Hereford	3	74681893	76225687	5	75725687^b^	5.73 × 10^–7^	0.005	0.003	0.976	0.000	0.995	0.390	CTH, LRRC7*, LRRC40
	5	79055337	80113473	8	79564409^a^	3.32 × 10^–7^	0.536	0.606	0.975	0.487	0.403	0.900	SINHCAF
	7	81624551	82816882	4	82124551^a^	1.88 × 10^–7^	0.994	0.003	0.995	0.000	0.003	0.991	TENM2, WWC1
	20	19842459	20942794	51	20401686^a^	2.44 × 10^–7^	0.043	0.098	0.266	0.271	0.198	0.146	PDE4D, RAB3C
	23	50140690	51876442	10	51357892^b^	8.96 × 10^–7^	0.277	0.755	0.229	0.786	0.184	0.582	SLC22A23, RIPK1, NQO2, GMDS*
Limousin	4	57644495	58664115	9	58148365^a^	5.52 × 10^–7^	0.974	0.053	0.932	0.092	0.953	0.335	IMMPL2
	6	31747431	35203508	1588	33609037^a^	1.17 × 10^–18^	0.249	0.879	0.415	0.151	0.260	0.812	SMARCAD1, ATOH1, CCSER1
	6	36934944	41871562	663	38035891^d^	1.45 × 10^–16^	0.086	0.000	0.000	0.128	0.636	0.007	PPM1K^, ABCG2^, PKD2^, SPP1^, MEPE*, LAP3, NCAPG^, LCORL^
	6	42312608	43680601	17	42990479^b^	1.48 × 10^–7^	0.000	0.006	0.000	0.029	0.000	0.000	ADGRA3, KCNIP4*
	11	104805923	105866536	3	105366536^b^	1.04 × 10^–7^	0.032	0.979	0.008	0.010	0.983	0.035	BRD3, WDR5, CACNA1B*
Simmental	8	82805400	83805881	3	83305881^a^	1.67 × 10^–6^	0.367	0.688	0.693	0.540	0.279	0.675	FANCC
	8	106857510	107869952	3	107357510^b^	8.48 × 10^–7^	0.990	0.073	0.928	0.093	0.859	0.878	PAPPA*, TRIM32
	12	55018060	56018149	3	55518060^a^	2.66 × 10^–7^	0.000	0.955	0.004	0.967	0.005	0.000	SPRY2
	12	89258864	90269817	3	89758864^a^	2.78 × 10^–6^	0.015	0.988	0.992	0.028	0.950	0.982	ANKRD10, ING1, SOX1, TUBGCP3
	22	1921471	3018467	32	2517667^a^	4.87 × 10^–7^	0.000	0.000	0.000	0.000	0.003	0.000	CMC1, AZI2
Holstein Friesian	4	108676456	109728131	8	109185322^a^	1.49 × 10^–6^	0.096	0.203	0.081	0.775	0.365	0.794	TPK1
	5	59814571	62558882	76	60701477^a^	4.28 × 10^–8^	0.257	0.900	0.953	0.874	0.949	0.894	NEUROD4, TSPA1, NTN4*, SNRPF*, AMDHD1*, LTA4H*, CDK17*, NEDD1
	5	104934097	106783101	135	106283101^a^	3.77 × 10^–8^	0.096	0.679	0.437	0.000	0.802	0.475	ANO2, NTF3, KCNA1, NDUFA9, FGF6*, FGF23*, TIGAR*
	6	60485248	61489096	26	60985248^d^	1.94 × 10^–6^	0.965	0.903	0.148	0.000	0.904	0.973	UBE2K, N4BP2, RHOH, CHRNA9*, RBM47
	7	23221527	24809431	46	23789810^b^	1.24 × 10^–7^	0.110	0.834	0.952	0.000	0.031	0.903	IRF1, PDLIM4, P4HA2, IL3, ACSL6, FNIP1*, HINT1

AA, Angus; CH, Charolais; HE, Hereford; LM, Limousin; SE, Simmental; HF, Holstein-Friesian. Superscript denotes SNP classification:^*a*^intergenic, ^*b*^intron, ^*c*^upstream gene variant, ^*d*^downstream gene variant. Symbols denote the significance of SNPs within genes: * gene contained at least one suggestive (*p* ≤ 1 × 10^–5^) SNP ^ gene contained at least one significant (*p* ≤ 1 × 10^–8^) SNP.

Of the 514 SNPs that were suggestively associated with stature in the HF, 281 were located on BTA5. Both the AA and HF had suggestive associations on this autosome; two intergenic SNPs, rs798298008 (AA) and rs475950607 (HF), located just 17 bp apart and 63 Kb from the *PTPRO* gene, were associated with WH in these breeds. The strongest associations in the remaining breeds were all intergenic SNPs, although their location differed by chromosome; the strongest association in the CH was on BTA2 in a 1 Mb QTL containing *MSTN*; the strongest association in the HE was in BTA7, with the strongest association for the SI located on BTA12.

There were 1,055 suggestive and 36 significant SNPs associated with WH in the meta-analysis (Supplementary Table S3). A single QTL on BTA15 containing multiple plausible candidate genes, such as *ALKBH8* and *RAB39A*, was the only QTL identified that had not previously been associated with WH in any of the within-breed analyses.

### Chest Width

The window-based analyses revealed no 1 kb genomic region suggestively associated with CW in more than one breed (Supplementary Figure S1). Similar to WH, BTA6 harbored the strongest QTL association with CW in the LM. This QTL, which also encompassed the *NCAPG*/*LCORL* complex, contained 34 suggestively associated SNPs, of which the strongest (rs110194711) was in the *MEPE* gene. A similar genomic region on BTA6 was also associated with CW in the HE, suggesting that the QTL region on BTA6 may harbor an across-breed pleiotropic association since it was also associated with WH in the AA and LM. Although four of the 6 breeds (AA, CH, HE, and HF) had QTLs on BTA10 suggestively associated with CW (Table 2 and Supplementary Figure S3), these all differed in their location across the chromosome which may suggest that BTA10 contains multiple genomic regions influencing CW.

**TABLE 2:** The location of the most significant QTLs, limited to the top 5, which were associated with chest width, and the genes located within these QTLs within each breed.

				**No of suggestive and significant SNPs**	**Most significant SNP**		**Allele frequency of positive allele**						
								
**Breed**	**Chr**	**Start**	**End**			***P*-Value**	**AA**	**CH**	**HE**	**LM**	**SI**	**HF**	**Candidate genes within this QTL**
Angus	8	19919026	20930648	3	20426751^b^	4.18 × 10^–6^	0.057	0.980	0.014	0.896	0.112	0.989	ELAVL2*
	10	101530896	102548539	4	102040999^b^	5.32 × 10^–7^	0.232	0.678	0.305	0.306	0.216	0.223	TTC8, FOXN3*
	11	52112729	53133828	13	52632756^a^	1.85 × 10^–6^	0.639	0.241	0.895	0.908	0.898	0.105	
	12	12006341	13006349	2	12506341^a^	2.69 × 10^–8^	0.073	0.047	0.943	0.000	0.918	0.963	VWA8, DGKH, TNFSF11, AKIP11
	28	2715813	4028680	11	3522966^d^	1.14 × 10^–6^	0.832	0.806	0.201	0.000	0.808	0.220	SPRTN, TRIM67*
Charolais	3	75099566	76200376	43	75636445^b^	2.40 × 10^–7^	0.111	0.071	0.000	0.000	0.903	0.168	CTH, LRRC7*, LRRC40
	9	12560255	13754168	9	13060255^a^	3.52 × 10^–7^	0.990	0.016	0.991	0.985	0.987	0.000	MTO1, EEF1A1
	10	42104985	43116388	3	42604985^a^	3.68 × 10^–7^	0.003	0.039	0.992	0.948	0.030	0.991	RPL36AL, MGAT2, ARF6, SOS2
	11	10962219	12944023	11	11462219^b^	3.04 × 10^–7^	0.000	0.007	0.000	0.000	0.000	0.000	ALMS1, EGR4, SMYD5, CYP26B1, SFXN5*
	18	57584619	58600780	3	58084619^d^	1.61 × 10^–7^	0.002	0.012	0.991	0.004	0.031	0.000	ENSBTAG00000014593*
Hereford	4	82061233	83396596	4	82561233^a^	2.12 × 10^–7^	0.000	0.989	0.023	0.012	0.994	0.993	POU6F2
	6	38955125	39995325	14	39461621^a^	6.63 × 10^–7^	0.308	0.000	0.852	0.000	0.000	0.604	LCORL
	7	79663134	80729587	3	80197062^a^	8.60 × 10^–9^	0.013	0.014	0.996	0.000	0.012	0.978	
	10	56443792	57546809	7	57025496^a^	9.84 × 10^–8^	0.961	0.000	0.967	0.050	0.893	0.062	WDR72
	23	8222426	9377363	9	8722426^d^	1.37 × 10^–7^	0.010	0.009	0.005	0.005	0.010	0.979	UHRF1BP1*, HMGA1, NUDT3, SCUBE3
Limousin	1	61741512	63549298	3	63048403^a^	1.08 × 10^–6^	0.439	0.000	0.650	0.730	0.449	0.225	
	6	37530341	38792617	34	38284104^b^	1.09 × 10^–7^	0.298	0.000	0.685	0.565	0.722	0.460	PPM1K, ABCG2*, PKD2*, SPP1, MEPE*, LAP3
	18	9391406	10382598	13	9891406^b^	1.69 × 10^–6^	0.211	0.249	0.109	0.137	0.447	0.136	CDH13*, HSBP1, MLYCD
	18	55221720	56247875	3	55721720^d^	1.39 × 10^–6^	0.995	0.000	0.002	0.003	0.000	0.000	LIG1, KCNJ14, CYTH2, RPL18, PPP1R15A
	20	7457546	8466248	16	7959103^a^	9.82 × 10^–7^	0.057	0.008	0.990	0.987	0.979	0.000	UTP15, ANKRA2
Simmental	13	70061402	71118226	7	70573855^a^	2.82 × 10^–6^	0.010	0.050	0.987	0.897	0.055	0.998	TOP1, PLCG1, LPIN3
	23	10151181	11174475	3	10651181^b^	1.20 × 10^–6^	0.071	0.913	0.054	0.128	0.068	0.959	CPNE5*, PIM1, TMEM217, TBC1D22B
	23	30033517	31047355	4	30533517^c^	1.67 × 10^–6^	0.099	0.072	0.219	0.187	0.902	0.146	ZSCAN31, ZKSCAN4, HIST1H2BB
	25	8699062	9699134	5	9199062^a^	6.83 × 10^–7^	0.000	0.000	0.996	0.000	0.005	0.996	EMP2, NUBP1, CLEC16A
	26	48445354	49451650	6	48945354^a^	2.03 × 10^–6^	0.989	0.008	0.996	0.041	0.996	0.000	
Holstein Friesian	1	57000435	58139976	15	57582901^b^	7.42 × 10^–7^	0.034	0.225	0.209	0.688	0.000	0.195	ABHD10, CD200*, ATG3, CCDC80
	2	30344158	31344250	3	30844250^a^	7.56 × 10^–7^	0.003	0.000	0.994	0.987	0.998	0.990	TTC21B, GALNT3, CSRNP3
	10	39919494	41220895	5	40476976^a^	1.04 × 10^–6^	0.004	0.004	0.975	0.991	0.982	0.861	MDGA3*
	13	78475631	79544490	9	79027846^a^	9.15 × 10^–7^	0.059	0.098	0.045	0.765	0.840	0.073	SNAI1, UBE2V1, PTPN1
	24	827290	2268995	9	1331600^a^	4.86 × 10^–7^	0.064	0.067	0.986	0.000	0.924	0.013	PQLC1, KCNG2, NFATC1, ATP9B

AA, Angus; CH, Charolais; HE, Hereford; LM, Limousin; SE, Simmental; HF, Holstein-Friesian. Superscript denotes SNP classification:^*a*^intergenic, ^*b*^intron, ^*c*^upstream gene variant, ^*d*^downstream gene variant. Symbols denote the significance of SNPs within genes: * gene contained at least one suggestive (*p* ≤ 1 × 10^–5^) SNP ^ gene contained at least one significant (*p* ≤ 1 × 10^–8^) SNP.

The meta-analysis of all 23,943 animals failed to identify a genomic region significantly associated with CW, but 170 SNPs were suggestively associated (Supplementary Table S3). The majority of these associations were singular SNPs, although peaks of suggestive association were detected on BTA1, BTA2, BTA8, BTA16, and BTA19.

### Hip Width/Rump Width

There were no 1 kb suggestive windows common to any of the breeds associated with width of hips. The QTL on BTA6 surrounding the *NCAPG* and *LCORL* genes was again significant in the LM although it failed to reach significance in the remaining five breeds (Table 3 and Supplementary Figure S4). Of the 222 SNPs suggestively associated with HW in the HE population, 52% were located in a QTL on BTA4 surrounding the *CLEC5A* gene. Although *MSTN* may have been expected to influence HW in the CH, the QTL on BTA2 associated with HW was located much further down-stream, between 30.21 and 31.26 Mb (Table 3). Several plausible candidate genes were located within this QTL on BTA2 including multiple voltage−gated sodium−channel genes, *TTC21B*, and *CSRNP3*; nonetheless only 0.07% of the genetic variation in HW was explained by the strongest association within this QTL. In the HF, the most significant SNP associated with RW was an intergenic SNP, rs382714953 (2.03 × 10^–7^), located on BTA20.

**TABLE 3:** The location of the most significant QTLs, limited to the top 5, which were associated with hip width or rump width, and the genes located within these QTLs within each breed.

				**No of suggestive and significant SNPs**	**Most significant SNP**		**Allele frequency of positive allele**						
								
**Breed**	**Chr**	**Start**	**End**			***P*-Value**	**AA**	**CH**	**HE**	**LM**	**SI**	**HF**	**Candidate genes within this QTL**
Angus	4	115417450	116432669	15	115922671^b^	6.53 × 10^–7^	0.031	0.925	0.109	0.840	0.788	0.268	KMT2C, ACTR3B*,XRCC2, CCT8L2
	5	30902961	31924821	5	31402961^a^	2.79 × 10^–7^	0.002	0.992	0.978	0.006	0.002	0.990	RHEBL1, PRKAG1, WNT1, WNT10B, CCDC65
	11	81485390	82623280	5	81985390^b^	1.09 × 10^–6^	0.004	0.000	0.000	0.996	0.994	0.000	FAM49A*
	20	13855925	14889348	17	14374205^a^	1.16 × 10^–6^	0.004	0.021	0.013	0.000	0.016	0.002	TRIM23, ADAMTS6
	25	15156974	16246007	4	15656974^a^	5.63 × 10^–7^	0.003	0.983	0.011	0.973	0.974	0.000	XYLT1
Charolais	2	30205997	31264765	30	30705997^a^	2.83 × 10^–8^	0.978	0.993	0.995	0.008	0.007	0.267	GALNT3*, SCN1A, SCN2A, SCN3A, TTC21B, CSRNP3
	8	4328030	5328051	4	4828030^b^	1.09 × 10^–6^	0.000	0.005	0.004	0.028	0.026	0.997	GALNTL6*
	9	12598999	13731582	8	13113448^a^	6.49 × 10^–7^	0.990	0.024	0.010	0.009	0.011	0.027	MTO1, EEF1A1
	15	7774063	8881109	3	8274063^b^	2.59 × 10^–7^	0.000	0.004	0.000	0.005	0.998	0.000	ARHGAP42*
	28	5674318	6741712	5	6241712^c^	1.09 × 10^–6^	0.002	0.004	0.996	0.000	0.006	0.000	PCNX2*
Hereford	4	105760789	106772084	113	106265147^a^	2.78 × 10^–7^	0.596	0.432	0.695	0.000	0.572	0.521	TAS2R3, TAS2R4, TAS2R38
	8	4170402	5731161	6	4670402^b^	3.39 × 10^–6^	0.000	0.989	0.997	0.000	0.000	0.000	GALNTL6*, GALNT7
	13	53374292	54375561	4	53874292^a^	2.94 × 10^–6^	0.784	0.292	0.690	0.727	0.309	0.880	STK35, PDYN, SIRPA
	14	5352193	6396755	6	5852193^a^	4.29 × 10^–6^	0.000	0.000	0.986	0.000	0.000	0.000	COL22A1, FAM135B
	18	21513927	22756651	3	22256651^b^	3.63 × 10^–6^	0.983	0.006	0.008	0.993	0.991	0.040	CHD9, RBL2, RPGRIP1L*, FTO*, IRX3
Limousin	5	16612583	17626967	5	17112583^a^	4.66 × 10^–7^	0.030	0.000	0.008	0.983	0.994	0.066	
	6	32350666	34490506	812	33611754^a^	1.95 × 10^–9^	0.246	0.880	0.366	0.150	0.232	0.819	
	6	37341111	40835172	153	38030341^b^	1.55 × 10^–9^	0.084	0.000	0.000	0.126	0.366	0.006	ABCG2^, PKD2^, SPP1*, MEPE, LAP3, NCAPG*, LCORL*
	13	76534127	77546426	23	77045666^d^	4.01 × 10^–6^	0.962	0.000	0.988	0.041	0.023	0.101	NCOA3, SULF2
	21	38149733	39222453	23	38702258^a^	3.41 × 10^–7^	0.000	0.940	0.003	0.002	0.997	0.000	
Simmental	1	79028842	80104503	3	79590057^b^	1.77 × 10^–7^	0.022	0.040	0.000	0.040	0.005	0.027	LPP*
	10	86379935	87382277	3	86879935^c^	1.13 × 10^–6^	0.009	0.988	0.000	0.000	0.995	0.000	YLPM1, PGF, EIF2B2, MLH3, ACYP1, ZC2HC1C, NEK9, TMED10
	11	24184879	25302455	4	24684879^a^	1.36 × 10^–6^	0.000	0.000	0.000	0.000	0.998	0.006	PKDCC
	18	9064056	10795231	11	10281382^a^	3.42 × 10^–7^	0.000	0.000	0.000	0.000	0.986	0.040	CDH13*, OSGIN1, MBTPS1, DNAAF1, TAF1C
	22	25717794	30456249	16	29136317^a^	1.11 × 10^–6^	0.000	0.030	0.000	0.987	0.997	0.000	CHL1*, CNTN3, PDZRN3, GXYLT2
Holstein Friesian	1	8144528	9875908	27	9335614^a^	1.37 × 10^–6^	0.097	0.209	0.206	0.000	0.783	0.226	ADAMTS1, ADAMTS5, APP
	9	31692809	33191394	7	32273403^a^	3.55 × 10^–6^	0.005	0.021	0.006	0.973	0.030	0.995	MAN1A1*, ASF1A, CEP85L, PLN, SLC35F1
	13	78476376	79544490	4	78976376^a^	1.23 × 10^–6^	0.862	0.640	0.923	0.629	0.701	0.230	SNAI1, UBE2V1, PTPN1
	20	63192522	64260191	3	63722163^a^	2.03 × 10^–7^	0.003	0.025	0.000	0.023	0.995	0.995	TAS2R1, SEMA5A
	24	49503031	50528738	3	50024697^b^	5.55 × 10^–7^	0.081	0.901	0.938	0.066	0.023	0.936	ACAA2, MYO5B*, MBD1, CXXC1

AA, Angus; CH, Charolais; HE, Hereford; LM, Limousin; SE, Simmental; HF, Holstein-Friesian. Superscript denotes SNP classification:^*a*^intergenic, ^*b*^intron, ^*c*^upstream gene variant, ^*d*^downstream gene variant. Symbols denote the significance of SNPs within genes: * gene contained at least one suggestive (*p* ≤ 1 × 10^–5^) SNP ^ gene contained at least one significant (*p* ≤ 1 × 10^–8^) SNP.

In comparison to WH and CW, the lead variant within the top five QTLs associated with HW in the AA, CH, HE and SI breeds was near fixation (Table 3). All of the lead variants in the top five QTLs in the SI breed were close to the fixation for the positive (i.e., wider) allele in the SI and fixed for the negative (i.e., narrower) allele in the HE. In contrast, the frequency of the positive alleles for each of the lead variants identified in the LM population ranged from low to moderate.

In the meta-analysis of HW and RW, suggestively associated QTL were located on BTA11, BTA15, BTA18, and BTA23 (Supplementary Table S3); none of these QTL had been previously identified in the individual breed analyses but they contained multiple possible candidate genes including *FKBP1P*, *CDH13*, *HSPB1*, *DNAAF1*, and *PSMB9*.

### Back Length

The window-based analyses revealed that no 1kb genomic region was suggestively associated with BL in all breeds, but 40 1 kb windows on BTA6 surrounding the *NCAPG* and *LCORL* genes were suggestively associated with BL in both the AA and LM (Supplementary Figure S1). In total, 96 SNPs within a QTL spanning from 37.9 to 40.4 Mb on BTA6 were suggestively associated with BL in the AA, of which 12 SNPs were either intronic SNPs, or downstream or upstream variants of the *NCAPG* and *LCORL* genes. In the LM, the most strongly associated SNP, rs110343895 (*p* = 4.24 × 10^–13^), was an intronic SNP located within *NCAPG* (Table 4 and Supplementary Figure S5). In total, seven SNPs located within the *NCAPG* gene and 15 SNPs within the *LCORL* gene were suggestively associated with BL in the LM. Of the 33 potentially disruptive variants within the *NCAPG* and *LCORL* complex that were tested for association, six were segregating in the LM population but none were significant. LM animals that had at least one copy of the minor allele for the top three associated SNPs, rs465117501, rs378370406 or rs110343895, within the *NCAPG* and *LCORL* complex had a longer back, 0.37 (SE = 0.18) units longer on average, than those with two copies of the major allele.

**TABLE 4:** The location of the most significant QTLs, limited to the top 5, which were associated with back length, and the genes located within these QTLs within each breed.

				**No of suggestive and significant SNPs**	**Most significant SNP**		**Allele frequency of positive allele**					
								
**Breed**	**Chr**	**Start**	**End**			***P*-Value**	**AA**	**CH**	**HE**	**LM**	**SI**	**Candidate genes within this QTL**
Angus	6	37939769	40455422	70	38443019^a^	5.79 × 10^–7^	0.139	0.000	0.847	0.207	0.311	PKD2, SPP1, MEPE, LAP3, NCAPG*, LCORL*
	6	40762050	42494936	24	41262050^b^	8.44 × 10^–7^	0.032	0.003	0.000	0.000	0.000	SLIT2*, PACRGL, KCNIP4*
	9	11789073	12803143	4	12298383a	1.17 × 10^–6^	0.008	0.032	0.979	0.987	0.969	RIMS1, KCNQ5
	12	84208854	85283107	29	84720853^a^	6.13 × 10^–8^	0.949	0.000	0.013	0.035	0.981	
	13	68993173	70000878	3	69495192^a^	6.75 × 10^–7^	0.026	0.000	0.032	0.073	0.060	
Charolais	2	1	10036842	5525	6808074^a^	3.96 × 10^–48^	0.000	0.079	0.000	0.972	0.996	WDR75, ASNSD1^∧^, ARHGEF4^∧^, MYO7B^∧^, NAB1^∧^, MFSD6^∧^, MSTN^∧^, PMS1^∧^, ORMDL1^∧^, COL3A1^∧^, COL5A2^∧^, ANKAR^∧^, SLC40A1^∧^
	14	33353270	34356964	4	33853270^a^	1.19 × 10^–7^	0.000	0.013	0.000	0.000	0.992	ARFGEF1, CPA6, PREX2
	14	44425358	45430890	3	44928243^a^	7.51 × 10^–7^	0.209	0.273	0.605	0.423	0.423	STMN2, HEY1, MRPS28
	28	19217733	21371343	36	19836248^a^	1.01 × 10^–7^	0.418	0.784	0.575	0.583	0.626	NRBF2, REEP3*
	28	30350477	31864396	38	31332353^a^	6.88 × 10^–9^	0.450	0.859	0.629	0.426	0.629	KAT6B*, DUPD1, DUSP13, VDAC2
Hereford	4	1	910718	5	223774^a^	1.15 × 10^–6^	0.975	0.984	0.981	0.981	0.000	VSTM2A*
	4	37522586	38567213	13	38055263^a^	2.31 × 10^–6^	0.959	0.283	0.130	0.851	0.201	PCLO*
	8	85462715	87578203	16	86646431^a^	1.63 × 10^–6^	0.000	0.000	0.998	0.000	0.000	OGN, ASPN, ECM2, IPPK, BICD2, FGD3, NINJ1, BARX1*,PTPDC1*
	14	30747311	31758061	7	31247311^a^	3.41 × 10^–6^	0.429	0.333	0.485	0.636	0.648	BHLHE22, MTFR1
	18	29621954	30630622	5	30130622^a^	8.58 × 10^–7^	0.996	0.995	0.996	0.985	0.010	CDH8
Limousin	1	66063243	67175049	15	66587440^b^	2.16 × 10^–7^	0.002	0.030	0.983	0.997	0.018	GTF2E1, STXBP5L, POLQ*, FBXO40, HCLS1, GOLGB1
	3	24752329	26688150	3	26188150^d^	9.48 × 10^–7^	0.000	0.897	0.908	0.917	0.888	SPAG17*, WDR3, MAN1A2, VTCN1*, TRIM45, TTF2, CD101, PTGFRN
	6	32025422	34384319	1058	33661101^a^	5.14 × 10^–13^	0.753	0.904	0.407	0.142	0.259	ATOH1
	6	36996616	41253691	469	38792702^b^	4.24 × 10^–13^	0.097	0.000	0.105	0.091	0.000	ABCG2^∧^, PKD2^∧^, SPP1*, MEPE, LAP3, NCAPG^∧^, LCORL^∧^, SLIT2
	21	33476048	34502357	6	33999605^a^	1.55 × 10^–6^	0.006	0.017	0.005	0.017	0.005	CSPG4, SNX33, IMP3, PTPN9
Simmental	15	77047714	78087312	9	77558153^b^	5.09 × 10^–7^	0.811	0.000	0.270	0.000	0.264	DGKZ, ATG13, ARHGAP1, ZNF408, CKAP5*
	16	10050545	11308116	5	10550545^a^	6.88 × 10^–7^	0.000	0.000	0.000	0.000	0.981	
	17	62751558	63784022	12	63254862^b^	1.24 × 10^–6^	0.047	0.940	0.977	0.930	0.969	LHX5*, PLDB2, OAS2, OAS1Y, OAS1X
	20	43798108	44854685	5	44298108^a^	2.56 × 10^–6^	0.042	0.069	0.240	0.074	0.109	
	21	10803227	11841095	7	11303227^a^	2.88 × 10^–6^	0.998	0.012	0.980	0.006	0.994	NR2F2

AA, Angus; CH, Charolais; HE, Hereford; LM, Limousin; SE, Simmental; HF, Holstein-Friesian. Superscript denotes SNP classification:^*a*^intergenic, ^*b*^intron, ^*c*^upstream gene variant, ^*d*^downstream gene variant. Symbols denote the significance of SNPs within genes: * gene contained at least one suggestive (*p* ≤ 1 × 10^–5^) SNP ^∧^ gene contained at least one significant (*p* ≤ 1 × 10^–8^) SNP.

A QTL on BTA2 was significantly associated with BL in the CH; this QTL stretched 10 Mb and contained 1,765 significant and 3,760 suggestive SNPs (Table 4). Fifty significant and 12 suggestive SNPs within this QTL were located within the *MSTN* gene; these SNPs included the well-known Q204X stop-gain mutation, rs110344317 (*p* = 2.01 × 10^–35^). When Q204X was forced into the model as a fixed effect, the most significant of the remaining SNPs on BTA2 generally reduced in significance relative to when Q204X was not included in the model. The most significant SNP on BTA2 after accounting for the variability in the Q204X genotype was rs41638272, an intergenic SNP located 15 kb from the *SLC40A1* gene. The QTL associated with BL also overlapped the QTL on BTA2 associated with WH suggesting this QTL may play a major role in affecting the morphology of an animal. No other significant associations with BL were identified in any of the remaining beef breeds.

In the meta-analysis of BL, significantly associated QTLs were identified on BTA2 and BTA6, similar to what was identified in the CH and LM breeds, respectively (Supplementary Table S3). Other QTLs on BTA12 and BTA13 were also associated with BL; the QTL on BTA13 contained numerous possible candidate genes including *DNTTIP1*, *TNNC2*, *PLTP*, and *CDH22* while no obvious candidate genes were identified on BTA12.

### Chest Depth

No suggestive or significant 1kb window associated with CD was common to more than one breed (Supplementary Figure S1). Only a single QTL on BTA6 containing the *NCAPG* and *LCORL* genes in LM was significantly associated with any of the breeds for CD (Table 5 and Supplementary Figure S6), suggesting that CD has a highly polygenic architecture in the beef breeds. Four of the five lead variants identified within the top five QTLs associated with CD in the AA were near fixation for the negative (i.e., narrower) allele while four of the five lead variants associated in the SI were close to fixation for the positive (i.e., deeper) allele. Only 90 SNPs were suggestively associated with CD in the CH, of which 19 were located on BTA10, but the proportion of genetic variance accounted for by the strongest association on this autosome was minimal (0.001%).

**TABLE 5:** The location of the most significant QTLs, limited to the top 5, which were associated with chest depth, and the genes located within these QTLs within each breed.

				**No of suggestive and significant SNPs**	**Most significant SNP**		**Allele frequency of positive allele**					
								
**Breed**	**Chr**	**Start**	**End**			***P*-Value**	**AA**	**CH**	**HE**	**LM**	**SI**	**Candidate genes within this QTL**
Angus	4	109535218	110566320	118	110035226^a^	2.08 × 10^–7^	0.003	0.000	0.000	0.000	0.998	CNOT4*
	8	51491571	52874502	6	52374502^b^	1.55 × 10^–7^	0.004	0.011	0.998	0.990	0.000	OSTF1, PCSK5*
	18	42431986	42811277	6	41931986^a^	8.41 × 10^–8^	0.004	0.003	0.000	0.014	0.996	
	19	25487490	26528596	129	25988404^b^	2.68 × 10^–7^	0.003	0.953	0.003	0.977	0.076	PITPNM3*, UBE2G1, MYBBP1A, GGT6, PIMREG
	23	27713725	28798254	34	28273994^c^	6.31 × 10^–7^	0.043	0.104	0.023	0.023	0.904	MIC1, TCF19, CCHCR1, VARS2, PPP1R18, TRIM26, TRIM15, TRIM10, TRIM40, TRIM31, TRIM39*, PPP1R11
Charolais	4	103847357	105940963	3	104347357^b^	2.48 × 10^–6^	0.000	0.006	0.000	0.993	0.005	HIPK, SLC37A3, WEE2, SSBP1, PARP12*
	10	29295461	30295461	12	29796031^b^	4.91 × 10^–6^	0.808	0.756	0.252	0.672	0.000	TMCO5B, SCG5
	10	75515119	76535772	7	76015119^b^	1.17 × 10^–6^	0.006	0.995	0.995	0.980	0.987	KCNH5, PPP2R5E*, SYNE2
	12	81616525	82648669	15	82139001^a^	4.14 × 10^–6^	0.053	0.100	0.000	0.027	0.921	NALCN, ITGB1
	14	49295193	50325837	6	49825837^b^	1.24 × 10^–6^	0.916	0.795	0.285	0.185	0.860	UTP23, EIF3H*
Hereford	3	63308338	64320629	4	63808996^a^	1.19 × 10^–6^	0.990	0.000	0.038	0.063	0.919	
	5	99016506	100071368	31	99516506^a^	6.26 × 10^–7^	0.100	0.056	0.070	0.966	0.046	
	17	61625220	62663494	3	62157617^a^	1.37 × 10^–6^	0.000	0.003	0.969	0.000	0.000	TBX3, TBX5
	18	41115715	42140232	4	41635699^a^	3.00 × 10^–6^	0.997	0.014	0.002	0.997	0.000	ZNF536, TSHZ3
	20	9677922	10679487	5	10177922^b^	2.93 × 10^–6^	0.863	0.257	0.741	0.666	0.219	MCCC2, BDP1, SERF1A, SMN2, SLC30A5
Limousin	5	26076148	27084460	3	26576148^c^	8.02 × 10^–7^	0.000	0.004	0.007	0.009	0.000	HOXC4, HOXC5, HOXC6, HOXC8, HOXC8, HOXC9, HOXC10, HOXC11, HOXC12, HOXC13
	6	32350666	34308736	456	33560360^a^	2.14 × 10^–7^	0.060	0.049	0.053	0.097	0.968	
	6	37037069	40568831	211	38075438^b^	2.92 × 10^–9^	0.087	0.000	0.000	0.131	0.368	PPM1K, ABCG2^∧^, PKD2^∧^, SPP1, MEPE, LAP3, NCAPG*, LCORL*
	7	16966648	17927749	15	17466648^a^	5.13 × 10^–7^	0.991	0.956	0.978	0.052	0.941	EBF1*
	11	77828096	78855720	3	78355720^a^	5.74 × 10^–7^	0.000	0.000	0.000	0.003	0.000	GDF7, RHOB, SDC1
Simmental	2	97634951	98536954	3	98035848^b^	2.77 × 10^–7^	0.000	0.002	0.000	0.000	0.004	KANSL1L, ACADL, MYL1
	11	42337336	43357452	3	42837336^a^	4.45 × 10^–7^	0.865	0.815	0.000	0.975	0.991	BCL11A, GTF2A1L*
	21	50755259	51864196	11	51364196^a^	4.44 × 10^–8^	0.000	0.002	0.000	0.002	0.998	LRFN5
	24	49238747	50334349	12	49739134^d^	4.03 × 10^–7^	0.997	0.002	0.005	0.005	0.995	CDH2*, DYM, ACAA2, MYO5B
	27	9276392	10276408	3	9776396^a^	3.29 × 10^–7^	0.000	0.007	0.000	0.975	0.998	

AA, Angus; CH, Charolais; HE, Hereford; LM, Limousin; SE, Simmental; HF, Holstein-Friesian. Superscript denotes SNP classification:^*a*^intergenic, ^*b*^intron, ^*c*^upstream gene variant, ^*d*^downstream gene variant. Symbols denote the significance of SNPs within genes: * gene contained at least one suggestive (*p* ≤ 1 × 10^–5^) SNP ^∧^ gene contained at least one significant (*p* ≤ 1 × 10^–8^) SNP.

In the meta-analysis, three SNPs were identified to be significantly associated with CD while 249 SNPs were suggestively associated. Three QTLs associated with CD in the meta-analysis were not significant in any of the single breed analyses and were located on BTA1, BTA5, and BTA13 (Supplementary Table S3 and Supplementary Figure S7).

### Across Trait Overlap

Quantitative trait loci associated with two or more skeletal traits were identified within each breed (Supplementary Figures S3, S8). The *NCAPG* and *LCORL* genes were identified as pleiotropic genes associated with all five traits in the LM breed and with both WH and BL in the AA breed. There were also suggestive genomic windows in common between CW and HW in the AA with five windows on BTA4 and a single window on BTA8 being common to both of these traits. These five windows on BTA4 contained six SNPs that were suggestively associated with both CW and HW; all six of these SNPs were intronic SNPs located within the *ENSBTAG00000008032* gene. No gene was located within the 1kb window on BTA8.

A greater overlap in QTLs associated with both WH and BL was identified in the CH and HE. Ten 1 kb windows were associated with both WH and BL in the CH, nine of which were located on BTA28. Eight 1 kb windows overlapped between WH and BL in the HE with 6 windows located on BTA23 encompassing the *GMDS* gene. Further overlap among traits was identified in the CH breed where three windows on BTA9 and three windows on BTA19 were associated with both WH and CW. The SI breed had the fewest number of pleiotropic associations of all beef breeds, as only one window on BTA12 near the *SPRY2* gene was suggestively associated with both WH and BL. The only overlap in associated QTLs between the beef and dairy breeds was in WH/Stature between the AA and HF. These breeds had two overlapping 1 kb windows on BTA5 but no obvious candidate genes were identified in this region.

### Enrichment of SNPs

Intergenic SNPs were the most common annotation class of SNPs associated with each trait in each breed. This annotation class was enriched for all traits in the HE, four traits in the LM (WH, BL, CD, and HW), three traits in the SI (WH, CW, and CD) and AA (WH, BL, and HW), and two in both the CH (WH and BL) and HF (CWD and RW; Table 6). The second most common annotation was the intronic SNPs; this class was enriched for three traits in the AA (CW, CD, and HW) and CH (BL, CW, and HW) and two traits in the SI (BL and HW). Downstream gene variants were enriched in all breeds for CW and at least one breed for all the remaining traits (Table 6). Stop-gain SNPs that were significantly associated with BL were enriched in all breeds in which they were associated.

**TABLE 6:** Fold enrichment/depletion of SNPs in each annotation class in each trait in each breed.

		**3′ UTR variant**	**5′ UTR variant**	**Downstream gene variant**	**Intergenic variant**	**Intron variant**	**Missense variant**	**Missense variant and splice**	**Non-coding transcript**	**Splice region variant**	**Stop gained**	**Synonymous variant**	**Upstream gene variant**
WH	AA	5.70	–	0.83	1.04	0.95	0.79	–	–	–	–	0.86	0.61
	CH	–	–	0.54	1.24	0.50	–	–	–	–	–	0.69	0.67
	HE	–	–	0.23	1.25	0.54	0.71	–	–	–	–	0.52	0.45
	LM	0.21	–	0.53	1.28	0.39	–	–	1.29	0.66	–	0.54	0.79
	SI	1.11	–	0.31	1.10	0.92	0.78	–	–	–	–	1.14	0.37
	HF	4.41	–	3.20	0.93	0.82	2.32	–	–	–	–	1.68	1.37
BL	AA	–	–	0.53	1.09	0.87	1.65	–	–	–	–	0.80	0.77
	CH	2.94	0.41	1.21	1.00	1.04	0.43	–	–	–	6.35	1.18	0.41
	HE	3.80	4.37	1.22	1.02	0.91	1.97	–	–	–	68.90	0.48	0.82
	LM	–	1.26	0.58	1.26	0.44	0.95	7.85	1.67	0.85	–	0.42	0.72
	SI	–	–	1.09	0.76	1.56	2.70	–	–	–	135.73	0.99	1.16
CW	AA	–	–	1.11	0.99	1.12	–	–	–	–	–	1.11	0.36
	CH	–	5.66	1.33	0.87	1.36	1.71	–	–	–	–	1.90	0.41
	HE	1.20	–	2.22	1.16	0.46	–	–	–	–	–	0.61	0.98
	LM	–	–	2.25	0.83	1.36	–	–	–	–	–	2.26	0.36
	SI	2.84	–	2.08	0.96	0.99	–	–	–	–	–	–	0.93
	HF	–	–	3.85	1.09	0.44	–	–	–	–	–	–	1.06
CD	AA	–	–	1.15	0.82	1.41	1.43	–	12.15	9.56	–	6.21	0.56
	CH	–	–	1.23	0.96	0.96	–	–	–	–	–	1.39	2.08
	HE	–	–	1.11	1.21	0.46	–	–	9.67	–	–	0.83	0.98
	LM	–	–	0.39	1.31	0.39	–	–	–	–	–	0.54	0.29
	SI	–	–	0.77	1.20	0.51	1.07	–	–	–	–	2.34	1.00
HW	AA	3.31	–	0.28	1.01	1.10	–	–	–	–	–	3.32	0.36
	CH	2.96	–	1.00	0.92	1.20	–	–	–	–	–	1.50	1.13
	HE	1.78	–	0.30	1.18	0.53	1.23	–	–	–	–	1.81	1.64
	LM	1.54	–	0.50	1.30	0.38	–	–	–	1.19	–	0.20	0.40
	SI	3.01	–	1.19	0.79	1.53	–	–	–	–	–	–	0.99
	HF	–	–	1.68	1.05	0.59	–	–	29.22	–	–	–	2.44

*AA, Angus; CH, Charolais; HE, Hereford; LM, Limousin; SE, Simmental; HF, Holstein-Friesian.*

## Discussion

Several QTLs were discovered in the present study to be associated with each of the skeletal type traits although the majority of these regions, excluding the *NCAPG*/*LCORL* locus in the LM population, were unique to a single trait or a single breed. This indicates the existence of breed-specific and trait-specific QTL for skeletal traits, which has implications for the usefulness of such QTL in across-breed genomic evaluations where only purebreds are used. Previous studies have documented both across-breed and breed-specific QTL associated with carcass traits, birth weight, weaning weight, and mature weight (Saatchi et al., 2014b), as well as dry matter intake, growth and feed efficiency (Saatchi et al., 2014a), carcass traits (Purfield et al., 2019), and muscular type traits (Doyle et al., 2020) in beef cattle. Excluding stature (Bouwman et al., 2018), the present study is the first published genome study on the skeletal linear type traits in beef cattle using imputed sequence data and is one of few genome-based studies comparing QTLs across multiple breeds of cattle. The present study, however, also incorporated imputed genome sequence information on 4,494 dairy cattle to compare to the beef animals. This comparison is rarely carried out (Purfield et al., 2015) as such multi-breed data are not always readily available for incorporation into the same study. Nonetheless, the difference in age at classification between the beef and dairy animals varied substantially with the beef animals all being < 16 months and the dairy animals > 23 months when assessed. Previous heritability estimates of the linear type traits assessed in the dairy cows were all ≥ 0.26 (Berry et al., 2004) indicating these traits are expected to be moderately to highly repeatable over time. This was substantiated by the fact that some common QTL were detected for Angus and Holstein-Friesian.

An earlier study on the beef cattle population from the dataset used in the present study (Doyle et al., 2018) summarized the heritability estimates of, and genetic correlations among, the skeletal type traits in each breed. In general, the genetic variance within each trait and the correlations between each trait differed by breed indicating that breed-specific and trait-specific QTL may be underlying these traits. Similarities were observed between the CH and LM in terms of heritability estimates and genetic correlations (Doyle et al., 2018); from this it was theorized that the genetic architecture of these breeds may be quite similar. The present study is an advanced version of this study (Doyle et al., 2018) where the contributors to the genetic variation within and across breeds have been identified.

Type traits have previously been proposed as potential early predictors of carcass weight and conformation (Conroy et al., 2010) and of overall carcass merit (Berry et al., 2019) given the genetic correlations between these traits and linear type traits are generally moderate to strong. However, as these correlations are not unity, two animals with the same live-weight may be morphologically very different which may lead to very different carcass value owing to the distribution of primal cuts (Berry et al., 2019). Therefore, type traits may be useful in future multi-trait genetic and genomic evaluations as they provide more information than live-weight alone. Consequently, knowledge of the QTLs associated with the skeletal traits could be used in these genome-based evaluations as part of a multi-trait evaluation targeting the altering of the morphology of an animal to increase the output of the goal trait (high quality primal cuts) thus improving the profitability of the farm system.

In total, over 90% of the QTLs identified in the present study have been previously documented to be associated with other production traits in beef or dairy cattle when compared to those within the Cattle QTLdb database (Accessed 08 January 2019). Of the top 140 QTLs associated with the skeletal type traits (Tables 2–6), 80 of these had previously been identified as being associated with body weight at either birth (Lu et al., 2013), as a yearling (Snelling et al., 2010), as a weanling (Saatchi et al., 2014b), at slaughter (Sherman et al., 2008), or at maturity (Saatchi et al., 2014b). Furthermore, some of the top 140 QTLs were also previously associated with carcass weight (McClure et al., 2010; Saatchi et al., 2014a) and residual feed intake (Nkrumah et al., 2007; Lu et al., 2013; Saatchi et al., 2014a) in cattle. Nineteen QTLs identified in the present study have also been identified previously as being associated with linear type traits describing the muscular characteristics of cattle (Doyle et al., 2020).

### Across-Breed Comparison

With the exception of the *NCAPG* and *LCORL* genes, the majority of QTLs associated with the skeletal type traits were breed-specific and in many cases, also trait specific. The differences observed in associated QTLs among the breeds may be due to epistatic or gene-by-environment interactions, or simply due to differences in the power to detect significance due to the large differences in population sizes among the breeds (Saatchi et al., 2014b). The age difference between the dairy and beef animals when classified may also have contributed to some of the inconsistencies in discovered QTL between the dairy and beef cattle. In many cases, the SNPs detected to associate with a trait in one breed were not segregating in all five breeds. Observed differences in detected QTL among the breeds may also be due to limitations in imputation where the imputed genotypes may not be perfect; this may result in the causal SNP not being identified as the most significant association especially if that SNP is rare in the populations (Bouwman et al., 2018).

Both *NCAPG* and *LCORL* are widely accepted as being associated with stature in many mammals including cattle (Bouwman et al., 2018), humans (Gudbjartsson et al., 2008), and horses (Tetens et al., 2013); therefore it was not unexpected that these genes were associated with all the skeletal traits in the LM population and with BL and WH in the AA. The *NCAPG* and *LCORL* genes have also been previously linked to growth and carcass traits in the SI (Zhang et al., 2018), carcass weight in the AA, CH, and LM (Purfield et al., 2019), and with both feed intake and body weight gain in a population containing 14 different breeds of cattle (Lindholm-Perry et al., 2011). Interestingly, the QTL containing *NCAPG* and *LCORL* was not associated with any of the skeletal traits evaluated in the SI or HF even though SNPs within these regions were segregating in both breeds. Although imputed sequence variants were used, we were unable to identify which of the two genes is causal; indeed none of the segregating missense variants within either gene were suggestively associated with any trait. However, a previous study that associated *LCORL* with growth and carcass traits in cattle, proposed that it is the non-coding and regulatory expression of *LCORL* that influences a trait (Han et al., 2017). This theory is further substantiated by the significant over-representation of the intergenic variant SNP class within the present study which suggests that it is the regulatory expression of many genes that influence animal morphology rather than the causative disruption of gene functionality.

### Carcass Traits

Some skeletal linear type traits in beef cattle are moderately genetically correlated with carcass traits including carcass cut weights (Pabiou et al., 2012), primal cut yields (Berry et al., 2019), and rib and subcutaneous fat thickness (Mukai et al., 1995). Thus, it is not surprising that there was overlap among some of the QTLs associated with linear type traits in the present study with those previously reported for carcass traits. Across all breeds and traits, there were 22 QTLs associated with the skeletal type traits in the present study that have been previously associated with carcass weight (McClure et al., 2010; Nishimura et al., 2012; Sharma et al., 2014). Twelve of these QTLs were located on BTA6 and incorporated the *NCAPG* and *LCORL* genes. Interestingly, the *NCAPG* and *LCORL* genes, while being associated with size have also been associated with subcutaneous fat thickness in beef cattle (Lindholm-Perry et al., 2011). More overlap among the QTLs associated with the skeletal type traits and fat thickness was on BTA2, where a QTL containing *MSTN* which was associated with BL and WH in the CH has also been documented to be associated with fat thickness at the 12th rib (Casas et al., 1998).

In general, if an allele was associated with a wider or longer skeletal type trait, it also had the same effect direction on the other traits, i.e., if an allele was associated with wider CW it tended to be associated with deeper CD and vice versa. Interestingly, this was not always the case for the alleles associated with WH and BL indicating that some alleles associated with taller WH were associated with shorter BL; thus, the correlation between these two traits (Doyle et al., 2018) could be broken leading to a morphologically different animal. The knowledge of SNPs and QTLs that influence one or more traits of interest (e.g., a longer back but with better muscling) would enable the selection for the desired trait combinations despite any genetic antagonisms. Furthermore, including traits such as WH and BL in a multi-trait genetic evaluation for terminal beef cattle, along with the other trait of interests (e.g., carcass weight, carcass conformation, and carcass fat) would provide more information on an animal’s carcass and conformation than what is possible from the carcass traits alone.

### Feed Intake and Efficiency

Feed intake is both genetically and phenotypically correlated with body weight and average daily gain (Arthur et al., 2001; Crowley et al., 2010); on average, bigger, heavier cattle tend to eat more. Feed is generally the greatest cost associated with beef production (Montano-Bermudez et al., 1990); thus, improvements in the efficiency of which feed is utilized should contribute to greater economic returns in the whole beef production system (Archer et al., 1999). Difficulty in selection for feed efficiency is mainly due to a lack of genetic evaluations for feed intake; data are generally readily available for the energy sink components of feed efficiency and thus selection for feed efficiency is being hindered by data on feed intake (or correlated traits). Feed intake is linked to the morphology of an animal (Crowley et al., 2011). While genomic evaluations for feed intake could be useful, the reference population required to generate accurate genomic evaluations are few. Having knowledge of potential QTLs associated with feed intake, discovered using much larger datasets on correlated traits (i.e., the present study), could be used as prior information in such genomic evaluations (MacLeod et al., 2016); the correlated traits could also be considered in a multi-trait genomic evaluation.

Among the QTLs associated with at least one of the skeletal type traits, 51 QTLs were previously identified as being associated with feed intake (Nkrumah et al., 2007; Sherman et al., 2010; Lindholm-Perry et al., 2011; Lu et al., 2013; Saatchi et al., 2014a) while 80 were previously identified as being associated with body weight at various stages of the animal’s life (Sherman et al., 2008; Snelling et al., 2010; Saatchi et al., 2014a) and body weight gain (Snelling et al., 2010). Given the generally small dataset sizes used in genomic analyses of feed intake traits, the QTL detected from the present study could actually be used as prior information in Bayesian-type analyses for genomic analyses (including genomic predictions) for traits like feed intake where the dataset size is limiting; such an approach could be deployed using models similar to those proposed by MacLeod et al. (2016).

### Calving Difficulty

The difficulty or ease of calving has long been thought to be related to the conformation of the dam (Ali et al., 1984) and the size of the calf (Sieber et al., 1989). Cows with wider hips and long rumps generally have larger internal pelvic openings which in turn lead to an easier calving; cows with smaller pelvic areas have more difficulty calving (Ali et al., 1984). Moreover, bigger, heavier calves are often more difficult to calve than their smaller, lighter counterparts (Sieber et al., 1989). It is, therefore, no surprise that 58 QTLs associated with the skeletal (i.e., size) type traits have previously been documented to be associated with calving difficulty in cattle (Purfield et al., 2015; Sahana et al., 2015). Seven of these 58 QTLs were associated with HW or RW in the present study; these QTLs were located on BTA1 in AA, BTA14 in HE, BTA6, BTA13, and BTA21 in LM, BTA10 in SI, and BTA1 in HF. None of the lead SNPs in these QTLs were segregating in all six breeds and a number of the lead SNPs were close to fixation for either the positive (i.e., wider hips) or negative (i.e., narrower hips) allele depending on the breed. Knowledge of the underlying quantitative trait variant associated with different morphological characteristics facilitates the development of more precise mating advice systems, over and above consideration of the holistic calving difficulty estimate breeding values based on genome-wide quantitative trait variants. For example, the choice of mate for a female with a genetic predisposition for a wide pelvic area is likely to differ from that of a female with a narrower pelvic area; knowledge of genetic merit of the mate for different skeletal characteristics, even with the same estimated breeding value for calving difficulty, should be exploited in the decision.

### Omnigenic Model of Complex Traits

It has long been hypothesized that many genes, each with a small effect size, underlie complex traits that do not exhibit simple Mendelian inheritance (Fisher, 1918). In recent years, and with the advancement of genomic technology, many studies have reported that even the most significant loci across the genome associated with a trait have small effect sizes and only explain a small percentage of the predicted genetic variance (Wood et al., 2014; Boyle et al., 2017). The term omnigenic has been used to describe the phenomenon whereby a very large number of genes with seemingly no relevance to the trait of interest are associated with that trait due to being in the same regulatory networks as the relevant genes (Boyle et al., 2017). The results of the individual genome-based analyses in the present study, where many SNPs of small effect, often located within regulatory regions were associated with each trait within each breed, confirms that a complex omnigenic genetic architecture underlies the skeletal type traits in the six cattle breeds.

Despite millions of SNPs being tested for associations with each of the skeletal traits investigated, only 140 of the SNPs suggestively or significantly associated with a trait were located within the coding regions of the genome. The majority (i.e., 57.2%) of SNPs associated with any trait were intergenic SNPs; the number of intergenic SNPs and also 3′ UTR and 5′ UTR variants were enriched for the majority of traits they were associated with in each breed, demonstrating the importance of regulatory networks within the genome to the cattle skeletal traits. Inference could also be drawn, therefore, on the contribution of regulatory regions to the correlated traits like carcass merit and feed intake. Downstream and upstream gene variants were also enriched in many of the traits. In general, the SNPs located within, or close to, the genes identified as candidate genes were located within these non-coding or regulatory regions. For example, 22 SNPs that were suggestively or significantly associated with WH in the LM were located within the *LCORL/NCAPG* gene; 19 of these were intronic variants and three were downstream gene variants. Thus regulatory non-coding regions, while not having an effect on the coding sequence of a gene, may be of particular importance for cattle skeletal development via the proposed omnigenic model (Boyle et al., 2017).

## Conclusion

While many QTLs were identified as being associated with each trait in each breed, a large-effect QTL on BTA6 containing the *NCAPG* and *LCORL* genes was the only QTL associated with more than two traits and in more than one breed. This indicates that while the *NCAPG* and *LCORL* genes may affect multiple traits in multiple breeds, the majority of QTLs underlying the skeletal type traits are both trait-specific and breed-specific. This has implications on the perceived usefulness of across-breed genomic evaluations for the component traits as well as possibly for their correlated economically important traits (e.g., carcass merit, feed intake) based solely on purebreds. Many of the QTLs identified in the present study have previously been documented to be associated with a number of other performance traits in cattle, including carcass traits, feed intake and calving difficulty.

## Data Availability Statement

Sequence variant genotypes were provided by participation in the 1000 Bulls Consortium and a subset of the sequences can be found at NCBI BioProject PRJNA238491, PRJEB9343, PRJNA176557, PRJEB18113, PRNJA343262, PRJNA324822, PRJNA324270, PRJNA277147, PRJNA474946, and PRJEB5462.

## Ethics Statement

Ethical review and approval was not required for the animal study because as the data were obtained from the existing Irish Cattle Breeding Federation (ICBF) national database (http://www.icbf.com).

## Author Contributions

JD, DB, RV, and DP participated in the design of the study and were involved in the interpretation of the results. JD performed the analyses and wrote the first draft of the manuscript. All authors read and approved the final manuscript.

## Conflict of Interest

The authors declare that the research was conducted in the absence of any commercial or financial relationships that could be construed as a potential conflict of interest.

## Footnotes

1 http://www.icbf.com

2 https://www.animalgenome.org/cgi-bin/QTLdb/BT/index
