# Supplementary material for: Genomic Regions Associated With Skeletal Type Traits in Beef and Dairy Cattle Are Common to Regions Associated With Carcass Traits, Feed Intake and Calving Difficulty
Source: Front Genet. 2020 Feb 4;11:20. doi: 10.3389/fgene.2020.00020 (PMC7010604; doi:10.3389/fgene.2020.00020)
Supplement: Supplementary file 2 [file Data_Sheet_2.pdf]

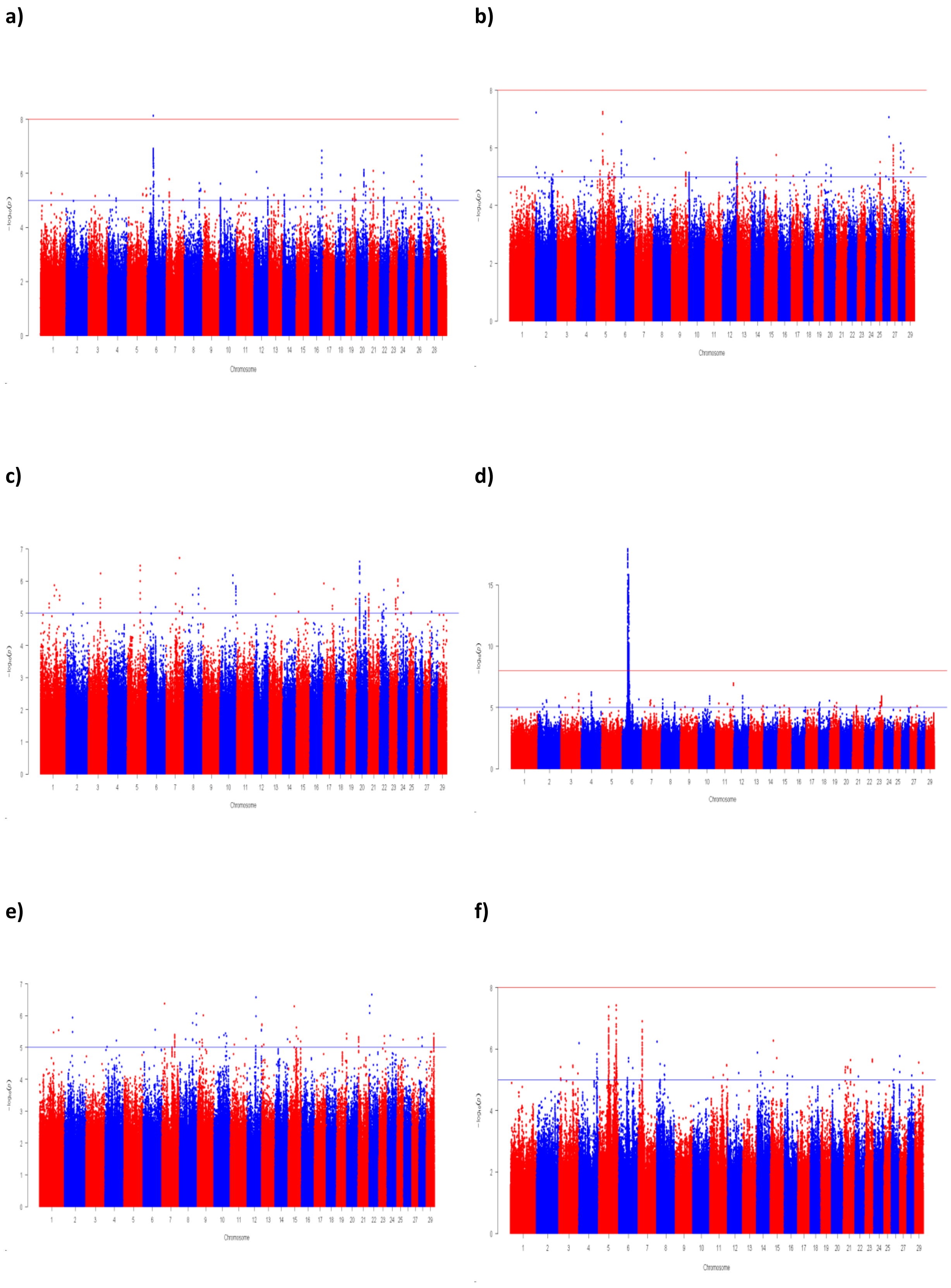

Figure S2: Manhattan plots for wither height in a) Angus, b) Charolais, c) Hereford, d) Limousin, e) Simmental, and f) stature in Holstein-Friesian.
