# Supplementary material for: Genomic Regions Associated With Skeletal Type Traits in Beef and Dairy Cattle Are Common to Regions Associated With Carcass Traits, Feed Intake and Calving Difficulty
Source: Front Genet. 2020 Feb 4;11:20. doi: 10.3389/fgene.2020.00020 (PMC7010604; doi:10.3389/fgene.2020.00020)
Supplement: Supplementary file 7 [file Data_Sheet_7.pdf]

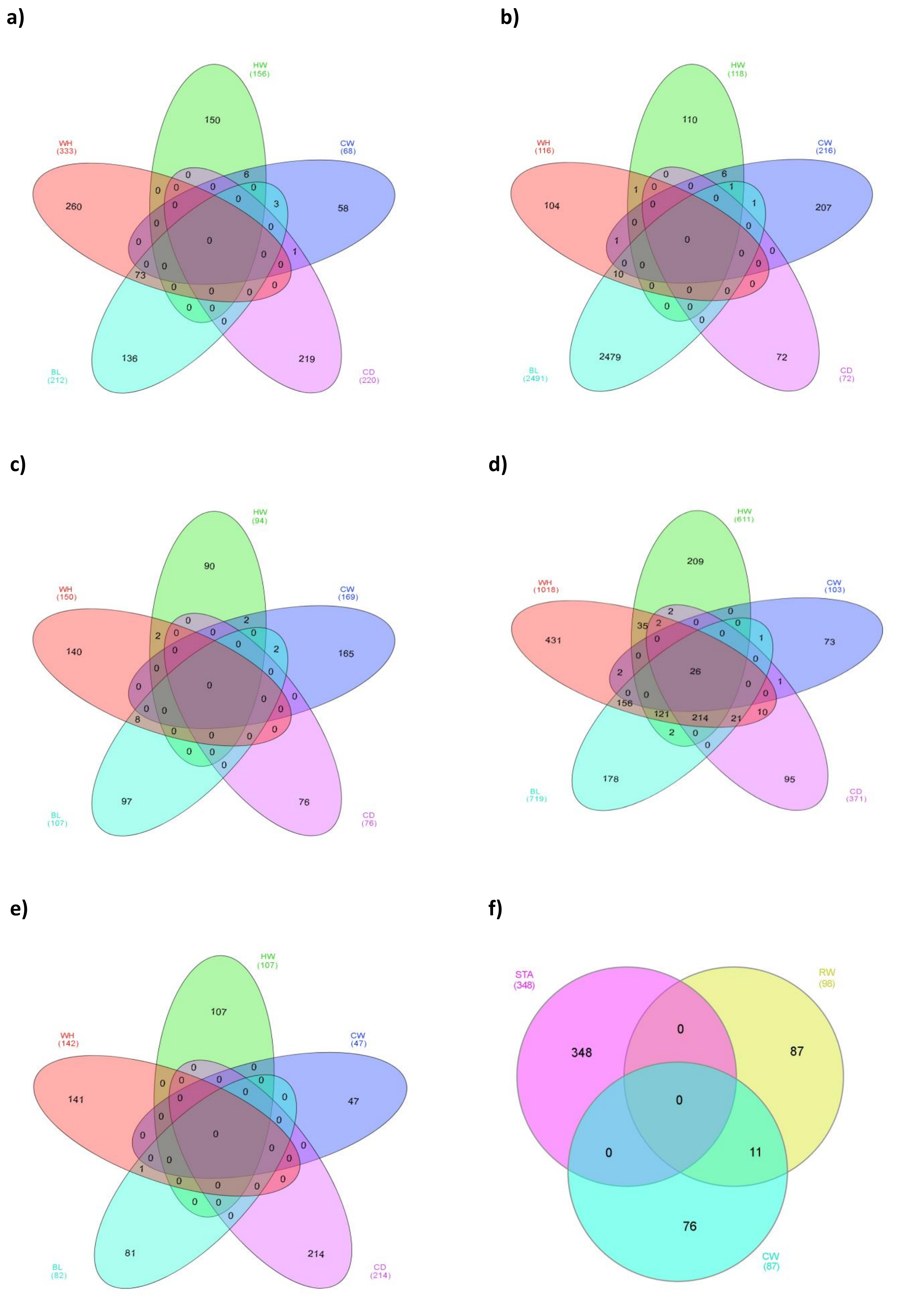

Figure S7: Overlapping 1kb regions that contain at least one suggestive or significant SNP for the 5 muscular traits in a) Angus, b) Charolais, c) Hereford, d) Limousin, e) Simmental, and three muscular traits in the Holstein-Friesian (f). The muscular traits were wither height (WH), hip width (HW), chest width (CW), chest depth (CD), and back length (BL)
