# Supplementary material for: Genomic Regions Associated With Skeletal Type Traits in Beef and Dairy Cattle Are Common to Regions Associated With Carcass Traits, Feed Intake and Calving Difficulty
Source: Front Genet. 2020 Feb 4;11:20. doi: 10.3389/fgene.2020.00020 (PMC7010604; doi:10.3389/fgene.2020.00020)
Supplement: Supplementary file 8 [file Data_Sheet_8.pdf]

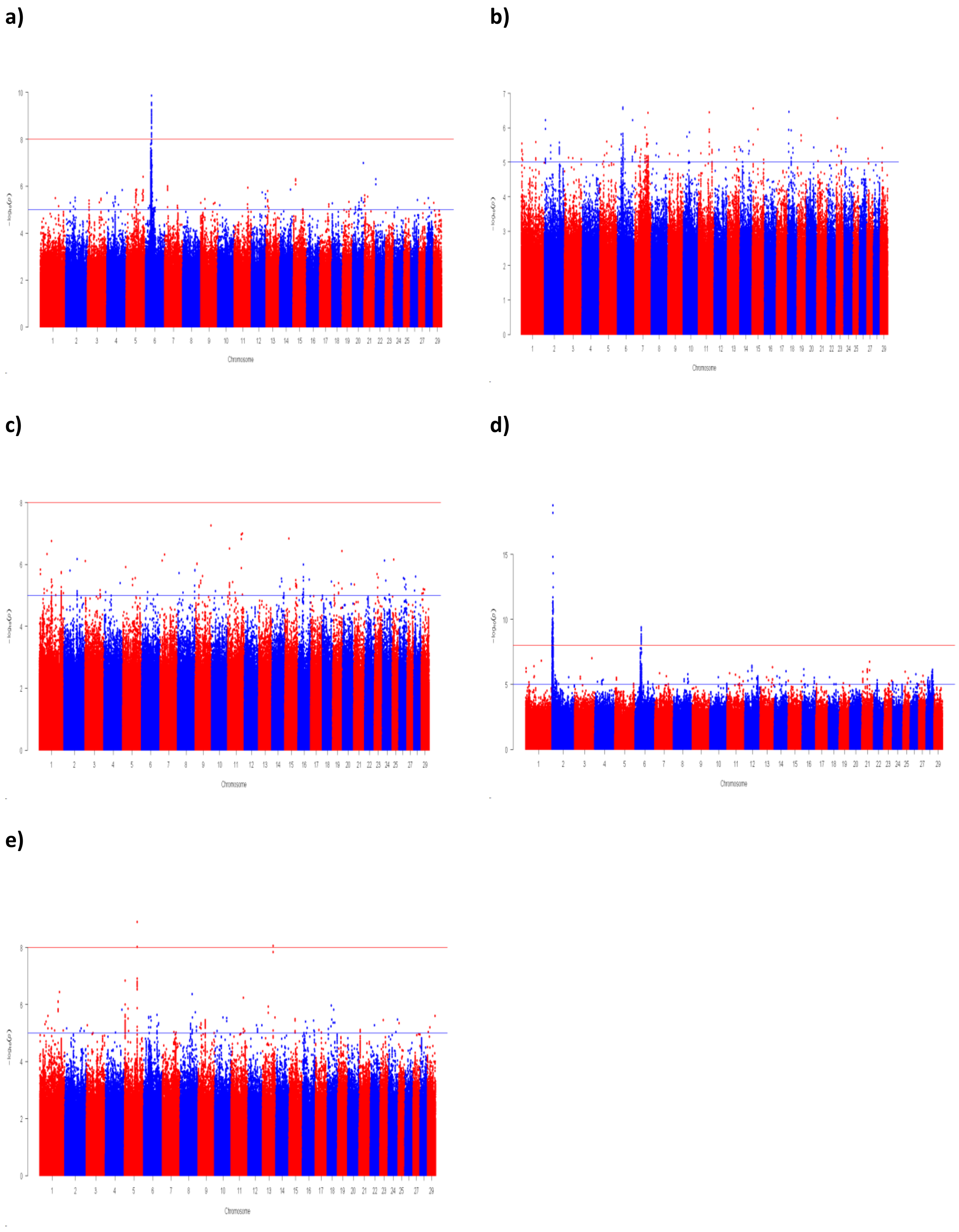

Figure S8: Manhattan plots for the meta-analysis in a) wither height, b) hip width, c) chest width, d) back length, and e) chest depth
