# Supplementary material for: Genomic Regions Associated With Skeletal Type Traits in Beef and Dairy Cattle Are Common to Regions Associated With Carcass Traits, Feed Intake and Calving Difficulty
Source: Front Genet. 2020 Feb 4;11:20. doi: 10.3389/fgene.2020.00020 (PMC7010604; doi:10.3389/fgene.2020.00020)
Supplement: Supplementary file 9 [file Table_1.pdf]

Table S1: The number of records, the mean, and the standard deviation of each linear type trait in each beef breed.

	Scale	Angus			Charolais			Hereford			Limousin			Simmental		
Trait	1-10	n	Mean	SD	n	Mean	SD	n	Mean	SD	n	Mean	SD	n	Mean	SD
Wither height	small - tall	1444	5.42	1.01	6432	5.98	1.13	1129	5.47	1.02	8745	5.74	1.09	1698	6.20	1.04
Chest width	narrow - wide	1434	5.63	0.96	6252	6.18	0.97	1128	5.64	0.88	8537	5.80	0.97	1619	6.06	0.95
Chest depth	shallow - deep	1433	6.35	0.90	6252	6.77	0.93	1128	6.36	0.88	8537	6.43	0.91	1619	6.84	0.87
Back length	short - long	1444	6.27	1.03	6432	6.75	1.07	1129	6.30	1.04	8745	6.59	1.08	1698	7.04	0.96
Hip width	narrow - wide	1444	5.46	0.95	6432	5.72	0.95	1129	5.66	0.89	8745	5.62	1.06	1698	5.96	0.95
