# Supplementary material for: Genomic Regions Associated With Skeletal Type Traits in Beef and Dairy Cattle Are Common to Regions Associated With Carcass Traits, Feed Intake and Calving Difficulty
Source: Front Genet. 2020 Feb 4;11:20. doi: 10.3389/fgene.2020.00020 (PMC7010604; doi:10.3389/fgene.2020.00020)
Supplement: Supplementary file 10 [file Table_2.pdf]

Table S2: The number of records, the mean, and the standard deviation of each linear type trait in Holstein Friesian.

		Holstein Friesian		
Trait	Scale 1-9	n	Mean	SD
Stature	small- tall	4494	5.96	1.47
Chest width	narrow - wide	4494	5.15	1.48
Rump width	narrow - wide	4494	5.49	1.42
