# Supplementary material for: Genomic Regions Associated With Skeletal Type Traits in Beef and Dairy Cattle Are Common to Regions Associated With Carcass Traits, Feed Intake and Calving Difficulty
Source: Front Genet. 2020 Feb 4;11:20. doi: 10.3389/fgene.2020.00020 (PMC7010604; doi:10.3389/fgene.2020.00020)
Supplement: Supplementary file 1 [file Data_Sheet_1.pdf]

a)

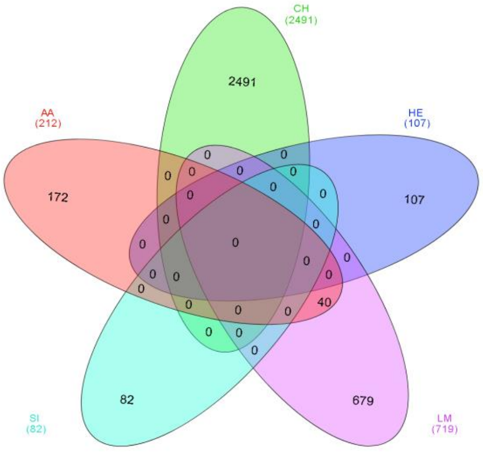

b)

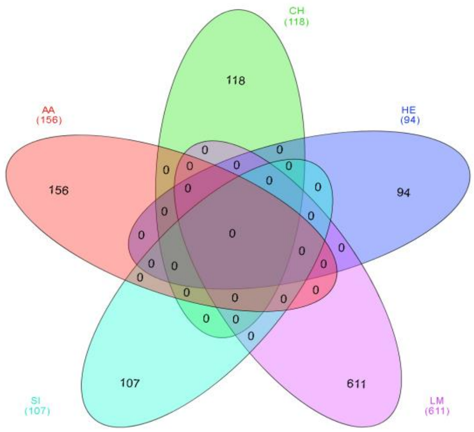

c)

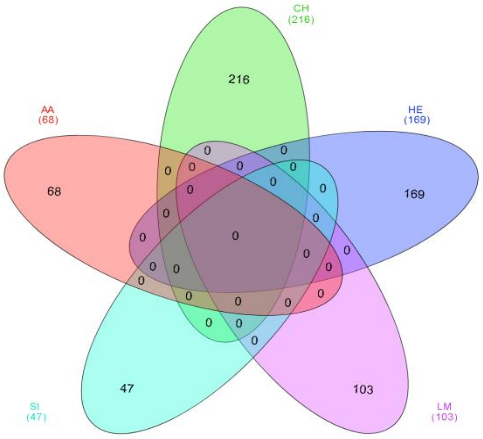

d)

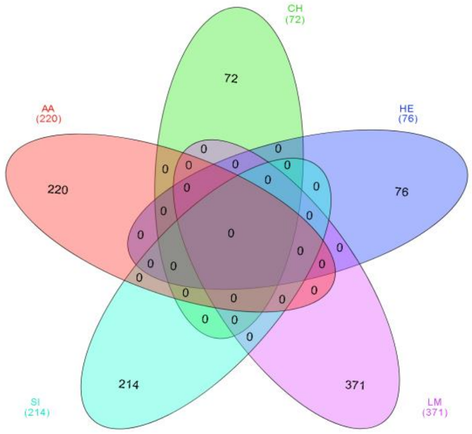

e)

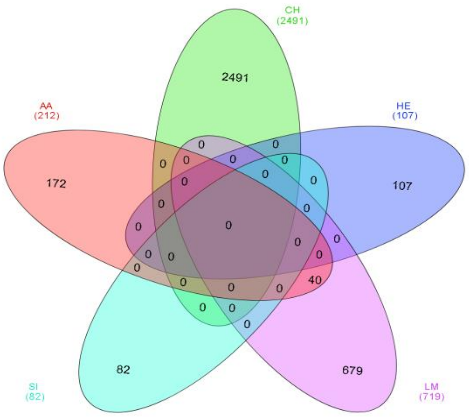

Figure S1: Overlapping 1kb regions that contain at least one suggestive or significant SNP in each breed for a) wither height, b) hip width, c) chest width, d) chest depth, and e) back length.
