# Supplementary material for: Genomic Regions Associated With Skeletal Type Traits in Beef and Dairy Cattle Are Common to Regions Associated With Carcass Traits, Feed Intake and Calving Difficulty
Source: Front Genet. 2020 Feb 4;11:20. doi: 10.3389/fgene.2020.00020 (PMC7010604; doi:10.3389/fgene.2020.00020)
Supplement: Supplementary file 11 [file Table_3.pdf]

Table S3: The location of the top 5 most significant QTLs associated with each of the skeletal traits in the meta-analysis.

| Trait | Chr | Start | End | No of suggestive and significant SNPs | Most significant SNP | P-Value | Candidate genes within this QTL |
|---|---|---|---|---|---|---|---|
| Wither Height | 5 | 105280734 | 106302598 | 28 | 105783088[a] | $4.00x10^{-7}$ | KCNA1, NDUFA9, FGF6, FGF23, TIGAR |
| | 6 | 32458499 | 35344463 | 590 | 33574963[a] | $1.60x10^{-8}$ | CCSER1 |
| | 6 | 36934944 | 40367479 | 233 | 38692833[a] | $1.39x10^{-10}$ | PPM1K, ABCG2, PKD2, SPP1, MEPE, LAP3, NCAPG, LCORL |
| | 15 | 16857667 | 17866073 | 3 | 17357667[a] | $5.08x10^{-7}$ | ALKBH8, RAB39A |
| | 22 | 1921471 | 3018467 | 32 | 2517667[a] | $4.87x10^{-7}$ | CMC1 |
| Chest Width | 1 | 78722748 | 79819964 | 9 | 79281613[b] | $1.78x10^{-7}$ | TPRG1, LPP |
| | 2 | 84546041 | 85547642 | 17 | 85047257[a] | $6.63x10^{-7}$ | SLC39A10, DNAH7, STK17B, HECW2 |
| | 8 | 111704513 | 112713771 | 5 | 112207970[b] | $1.49x10^{-6}$ | CDK5RAP2, FBXW2, TRAF1 |
| | 16 | 34222491 | 35399155 | 7 | 34722616[b] | $1.01x10^{-6}$ | AKT3, SDCCAG8, CEP170 |
| | 19 | 60882962 | 61882988 | 3 | 61382974[a] | $3.64x10^{-7}$ | KCNJ2, KCNJ16, MAP2K6, ABCA5 |
| Chest Depth | 1 | 116367840 | 117409053 | 15 | 116884742[c] | $7.63x10^{-7}$ | MBNL1, AADAC |
| | 5 | 3711874 | 4919423 | 75 | 4314400[a] | $1.44x10^{-7}$ | CAPS2 |
| | 5 | 80773564 | 81859095 | 13 | 81273564[a] | $1.29x10^{-9}$ | CCDC91 |
| | 11 | 77828096 | 78855720 | 2 | 78355720[a] | $5.74x10^{-7}$ | GDF7, RHOB, SDC1 |
| | 13 | 67722025 | 68774518 | 3 | 68222752[b] | $8.81x10^{-9}$ | KIAA1755, ADIG, DHX35 |
| Back Length | 2 | 1 | 9238659 | 969 | 5535691[a] | $1.69x10^{-19}$ | ASNSD1,ARHGEF4,MYO7B,NABI,MFSD6,MSTN,PMS1,ORMDL1,COL3A1,COL5A2,ANKAR,SLC40A1 |
| | 6 | 32966339 | 34249299 | 192 | 33471768[d] | $1.66x10^{-8}$ | |
| | 6 | 36399608 | 40835172 | 107 | 38672441[c] | $4.04x10^{-10}$ | PPM1K, ABCG2, PKD2, SPP1, MEPE, LAP3, NCAPG, LCORL |
| | 12 | 41360854 | 42774040 | 8 | 41860854[a] | $3.67x10^{-7}$ | |
| | 13 | 75166652 | 76244228 | 3 | 75166652[b] | $4.86x10^{-7}$ | DNTTIP1, TNNC2, PLTP, NCOA5, CDH22, OCSTAMP |
| Hip Width | 6 | 37042897 | 40544352 | 36 | 38648218[b] | $2.58x10^{-7}$ | PPM1K, ABCG2, PKD2, SPP1, MEPE, LAP3, NCAPG, LCORL |
| | 11 | 74549091 | 75686706 | 7 | 75078608[b] | $3.54x10^{-7}$ | NCOA1, ITSN1, FKBP1B, ATAD2B, KLHL29 |
| | 15 | 7741333 | 8881109 | 3 | 8381102[a] | $2.76x10^{-7}$ | ARHGAP42 |
| | 18 | 9307588 | 10781382 | 4 | 10281382[a] | $3.42x10^{-7}$ | CDH13, HSBP1, MBTPS1, DNAAF1, TAF1C, ATP2C2, COTL1 |
| | 23 | 6956952 | 7970369 | 3 | 7456952[a] | $5.27x10^{-7}$ | GCLC, DSB, TAP2, PSMB8, TAP1, PSMB9, COL11A2, HSD17B8, RING1, RPS18, DAXX, BAK1 |

Superscript denotes SNP classification: [a]intergenic, [b]intron, [c]upstream gene variant, [d]downstream gene varianT
